# Predicting the Epidemiological Effects in the United Kingdom of Moving from PCV13 to PCV15 in the Routine Pediatric 1 + 1 Vaccination Schedule

**DOI:** 10.3390/vaccines13060627

**Published:** 2025-06-10

**Authors:** Rachel J. Oidtman, Natalie Banniettis, Jessica Weaver, Ian R. Matthews, Dionysios Ntais, Giulio Meleleo, Tufail M. Malik, John C. Lang, Oluwaseun Sharomi

**Affiliations:** 1 Merck & Co., Inc., Rahway, NJ 07065, USA; natalie.banniettis@merck.com (N.B.); jessica.weaver@merck.com (J.W.); tufail.malik@merck.com (T.M.M.); 2 MSD (UK) Ltd., Value, Access, and Devolved Nations (VAD), London EC2M 6UR, UK; ian.matthews@msd.com (I.R.M.); dionysios.ntais@msd.com (D.N.); 3 Wolfram Research, Inc., Champaign, IL 61820, USA; giuliom@wolfram.com; 4 Merck Canada, Inc., Kirkland, QC H9H 4M7, Canada; john.lang@merck.com

**Keywords:** dynamic transmission model, invasive pneumococcal disease, pneumococcal conjugate vaccine, pediatric immunization, United Kingdom

## Abstract

Background/Objectives: Pneumococcal conjugate vaccines (PCVs) were first introduced in the pediatric UK National Immunization Programme (NIP) in 2006 and subsequently led to a significant decline in invasive pneumococcal disease (IPD). In 2020, the UK NIP reduced the pediatric PCV dosing schedule from two infant doses and one toddler dose (2 + 1) to one infant dose and one toddler dose (1 + 1). This analysis evaluated the public health impact of pediatric vaccination with PCV15 versus PCV13 under a 1 + 1 schedule. Methods: A population-level compartmental model was previously adapted to the UK setting. The impact on the IPD incidence of vaccination with PCV15 versus PCV13 under a 1 + 1 schedule was evaluated over a 20-year time horizon. The uncertainty regarding the vaccine efficacy (VE) of PCV13 and PCV15 under a 1 + 1 schedule was investigated through a probabilistic sensitivity analysis, i.e., the PCV VE under a 1 + 1 schedule was assumed to be 0–24% lower than the PCV VE under a 2 + 1 schedule. Results: Relative to the initial IPD incidence, vaccination with PCV13 and PCV15 under a 1 + 1 schedule resulted in the IPD incidence in children <2 years old increasing by 11.1% (95% region: 8.4–14.5%) and 3.5% (0.2–7.7%), respectively, over the time horizon. At the end of the time horizon, in the overall population, PCV15 would lead to a 6.0% lower IPD incidence than PCV13 (10.70 IPD cases per 100,000 versus 11.38 per 100,000, respectively). Conclusions: Switching from PCV13 to PCV15 for routine pediatric vaccinations under the 1 + 1 dosing schedule in the UK led to a lower IPD incidence in both the pediatric and overall populations.

## 1. Introduction

Pneumococcal disease (PD), caused by *Streptococcus pneumoniae*, leads to significant morbidity and mortality, especially in young children [1]. Preventative pneumococcal conjugate vaccines (PCVs) targeting clinically relevant serotypes are recommended for routine administration in early childhood. PCV7 was initially licensed 25 years ago as a four-dose schedule, with three doses in infancy followed by a toddler dose (3 + 1) [1]. Based on immunogenicity data supporting reduced dosing schedules, coupled with economic pressures and crowded childhood immunization schedules, the United Kingdom (UK) became the first country to adopt PCV7 in a three-dose (2 + 1) schedule in their National Immunization Programme (NIP) in 2006 [2,3]. In the adult population, PPSV23 has been widely used for adults aged ≥65 years old since 2003 [4].

Before the introduction of PCVs in the UK, overall invasive pneumococcal disease (IPD) incidence was 14.8 per 100,000, with PCV7 serotypes accounting for approximately half of the IPD disease burden [4]. By 2010, the overall incidence of IPD had dropped to 10.1 cases per 100,000, with PCV7 serotypes comprising 14% of IPD [4]. However, a relative increase in the proportion of non-vaccine-type (NVT) PD led to the adoption of PCV13 in 2010, which covered 58% of the disease burden at that time [4]. By 2019–2020, the overall disease incidence reached a low of 9.4 per 100,000 and NVT IPD comprised about 81% of the disease burden [1]. The reduction in overall IPD, with NVT driving the residual burden of disease, motivated two changes for the pediatric PCV NIP—the move to a 1 + 1 dosing schedule, and interest in switching to a higher-valency PCV. These changes were further supported by several factors: (1) high vaccine coverage rates leading to good control of vaccine-type (VT) PD [3]; (2) a 2018 randomized control study by Goldblatt et al., which demonstrated similar immune responses to PCV13 for 2 + 1 and 1 + 1 dosing schedules [2]; and (3) a 2019 modeling study by Choi et al. projecting that the incidence of IPD and non-bacteremic pneumococcal pneumonia (NBPP) would not be affected by a reduction in dosing schedule [5].

The decision to move to a 1 + 1 dosing schedule was not without risk, as fewer doses in infancy, when the immune system is immature, may lead to subpar protection and breakthrough disease. A separate modeling study conducted by Wasserman et al. explored the projected potential effects of switching to a 1 + 1 PCV13 dosing schedule in the UK [6]. This study estimated that the reduction in the number of infant doses was projected to lead to an increase in IPD cases over a 10-year time horizon, with the greatest increases occurring in infants and older adults, also resulting in an increase in deaths [6]. This finding was consistent with the results of the randomized control study by Goldblatt et al., which, while supporting the switch to a 1 + 1 dosing schedule, also noted this potential risk [2].

In January of 2020, the 1 + 1 reduced dosing schedule was implemented in the UK, with the infant dose administered at 12 weeks of age and the toddler dose at 1 year [7]. Contagion control measures for the COVID-19 pandemic were implemented soon after this switch, rendering it difficult to estimate the effects of this policy change due to the overall reduction in PD. A real-world observational study, published in 2024, indicated that overall IPD incidence in 2022/23 decreased in the years since the implementation of the 1 + 1 dosing schedule (which included the period when COVID-19 pandemic measures were in effect) [1]. However, VT IPD incidence increased over this period, which may hint at the fact that the immune response for a 1 + 1 schedule is insufficient to maintain prior levels of control of VT IPD, though it is difficult to draw conclusions given the confounding effects of COVID-19 during this timeframe [1].

The percentages of PD due to VT and NVT in the UK are still substantial and warrant the consideration of higher-valency PCVs in the pediatric NIP. Due to the reduction in serotype-specific immunogenicity as more serotypes are added to a PCV (“immunogenicity-creep”), there is a risk of reduced effectiveness of higher-valency PCVs [8,9]. Nevertheless, the replacement of PCV7 with the higher-valency PCV13 in the UK did not lead to an increase in VT disease [4]. Two new expanded-valency PCVs were recently licensed in the UK—PCV15 (Vaxneuvance^TM^, Merck & Co., Inc., Rahway, NJ 07065, USA) and PCV20 (Prevnar 20^TM^, Wyeth Pharmaceuticals LLC, a subsidiary of Pfizer, Inc., New York, NY 10001-2192, USA)—that provide protection against additional NVT serotypes [1]. The clinical impact of these new PCVs and their potential for immunogenicity creep expected in 1 + 1 dosing remains to be seen.

The confluence of information from immunogenicity studies on reduced dosing schedules, epidemiological data, transmission models, and the impact of the COVID-19 pandemic on the epidemiology of infectious diseases paints a confusing picture of the potential effectiveness of a 1 + 1 dosing schedule for pediatric pneumococcal vaccination in the UK with PCV13, and potentially with higher-valency PCVs. This analysis employed a previously described and calibrated model to explore the potential impact of reduced vaccine effectiveness (VE) against disease resulting from a reduced dosing schedule, using pediatric vaccines PCV13 and PCV15.

## 2. Materials and Methods

### 2.1. Model Overview

The dynamic transmission model used in this analysis was a published deterministic, age-structured, population-level model accounting for (1) demographic components, including births, deaths, and aging, and (2) carriage transmission dynamics in the presence of historical vaccine introduction [10,11]. The demographic model assumed a constant population size and included age structure in the form of four age groups: <2-, 2–4-, 5–64-, and ≥65-year-olds [12,13]. Mixing among populations of different ages is particularly relevant for *S. pneumoniae* transmission and was accounted for with a previously published mixing matrix [14].

**Figure A1:**
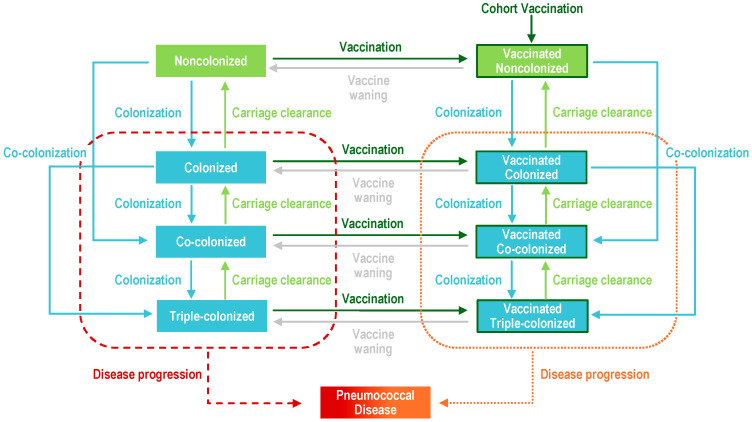
Model diagram. Upon carriage acquisition, individuals moved to either the colonized, co-colonized, or triple-colonized classes, depending on whether one or two serotypes were acquired (denoted in blue). Some portion of individuals from these colonized classes would develop a pneumococcal disease (denoted in red and orange). Recovery then moved individuals back to the non-colonized class (denoted in light green), sequentially clearing one serotype at a time. Vaccination (dark green border) reduced the risk of carriage acquisition and disease development for vaccine serotypes.

The epidemiological model was based on a system of ordinary differential equations and distinguished between pneumococcal carriage (single, double, and triple) and disease, as well as vaccination status. The progression from pneumococcal carriage to pneumococcal disease was modeled through an age-, serotype class- (STC), and vaccine-status-specific relationship, wherein disease occurred in some fraction of carriage episodes (Appendix A Figure A1). Given the relative infrequency of transmission from adults to children, the simplifying assumption was made that no transmission occurred from adults to children [15]. For the purpose of this model, the pediatric age strata comprised <2- and 2–4-year-olds and the adult age strata comprised 5–64- and ≥65-year-olds.

A complete description of the model, inputs, parameter estimates, and calibration results is provided by Oidtman et al. 2025 [11]. In brief, the model was calibrated using the Nelder–Mead simplex method implemented in the NMinimize function in Mathematica 13.3.1 (Wolfram Research, Champaign, IL, USA) to minimize a weighted sum of squared errors objective function over the annual age- and STC-specific IPD incidence data [16]. During the calibration, four sets of age- and STC-specific parameters were estimated: vaccine efficacy against carriage, carriage acquisition rate given contact, invasiveness (case-to-carrier ratio), and pairwise competition between STCs [11]. For the competition parameters, historical replacement dynamics were used to calibrate the specific STC-STC combinations. In the absence of observed data on replacement dynamics, as is the case between historical novel VTs in PCV13 and the novel VTs in PCV15, there was assumed to be no explicit competition.

### 2.2. Inputs and Data Sources

Annual age- and serotype-specific IPD incidence data for 2000–2019 were provided by the UK Health Security Agency (HSA) [4] and were used as a model calibration target for the pre-PCV NIP era steady state (2000–2005) and the vaccine period (2006–2019). As the model is driven by carriage dynamics, *S. pneumoniae* carriage data from 2006 were used as a baseline target for the pre-PCV NIP era [17,18]. Serotype-specific IPD and carriage data were aggregated into 11 STCs based on the inclusion of different serotypes in different vaccines to calibrate to historical data (Table 1) [19,20].

Serotype- and age-based data on VE against IPD were available for PCV13 [22] and PPSV23 [23]. Given the lack of real-world evidence for VE against IPD for novel PCV15 serotypes, VE was estimated as the weighted average (weighted by the number of STs in each PCV grouping) from prior vaccine-specific estimates (Appendix A Table A1 and Table A2). The base case values for VE against IPD (Table 2) were calculated by applying a 12.8% average reduction in the PCV13 VE under a 2 + 1 schedule, based on an observational study by Savulescu et al. [22]; additional details are provided in the Sensitivity Analysis subsection. Data on serotype-specific carriage clearance rates (i.e., the inverse of the duration of carriage) were available from studies from multiple countries and settings [24,25,26,27,28,29,30,31,32,33,34,35,36], which were averaged over serotype and age to obtain aggregate inputs into the model (more details are available in Appendix A Table A6). Data on vaccination coverage rates by age were available from the HSA (Appendix A Table A3, Table A4 and Table A5) [37].

### 2.3. Projection Scenarios

The calibrated model was used to evaluate the impact of implementing a 1 + 1 dosing schedule among children <2 years old with either PCV13 or PCV15, beginning in 2020. Both scenarios assumed a continuation of PPSV23 in older-adult and risk-group populations. In both pediatric and adult populations, it was assumed that vaccine coverage rate (VCR) levels from 2019 remained constant into the projected 20-year time horizon (Appendix A Table A3, Table A4 and Table A5). Reductions in pneumococcal disease or contact patterns arising from protection measures enacted during the COVID-19 pandemic were not accounted for in these projections. The model projection scenarios are summarized in Table 3.

### 2.4. Sensitivity Analysis

To explore the uncertainty in the potential reductions in VE against IPD associated with a reduced pediatric dosing schedule, a probabilistic sensitivity analysis (PSA) was performed, which varied the vaccine effectiveness against IPD for both PCV13 and PCV15. This analysis did not consider potential reductions in vaccine efficacy against carriage.

Potential reductions in VE against IPD resulting from the adoption of a reduced dosing schedule were informed by the estimated 12.8% average reduction in PCV13 VE when shifting from a 3 + 1 schedule to a 2 + 1 schedule, based on an observational study by Savulescu et al. of PCV13 VE against IPD in pediatric populations in Europe [22]. The range in VE values for the PSA was defined as follows: the upper limit was set to equivalency, such that the VE would be equivalent in a 1 + 1 and 2 + 1 dosing schedule; the mean value was set to the average 12.8% reduction following Savulescu et al. [22]; and the lower limit was set to a maximum reduction informed by doubling the average reduction (Table 2).

The VEs were drawn from beta distributions with ranges approximately equal to those described in Table 2, and the parameters were varied in a Latin hypercube sampling method with a total of 100 random samples [38]. The PSA did not consider differences in VE by vaccine; for example, a reduced VE in PCV7 STs was applied equally to both PCV13 and PCV15.

## 3. Results

### 3.1. Model Calibration

**Figure A2:**
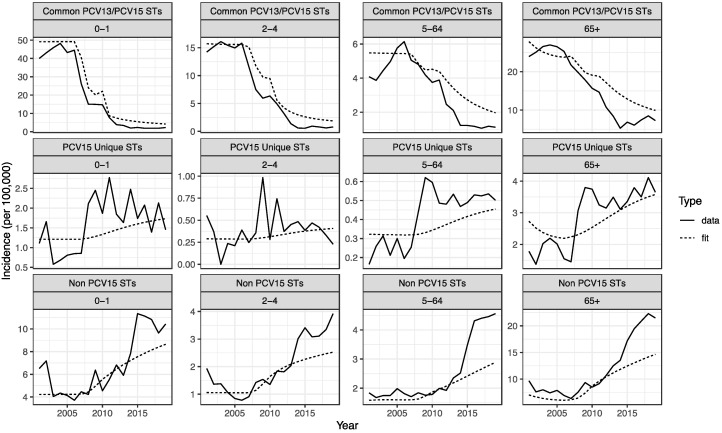
Model calibration to historical observed IPD incidence per 100,000 people by ST grouping and age group. Comparison of data (solid line) to model fit (dotted line) during the calibration period, faceted by age group and vaccination grouping.

The model was able to closely reproduce historically observed IPD dynamics, including the decline in incidence of VT IPD following the introduction of PCV7 and PCV13 and the subsequent increase in NVT IPD due to serotype replacement. In the <2- and 2–4-year-old populations, there were some observed dynamics that the model was not able to completely reproduce, such as the large increases in non-PCV15 serotypes beginning in 2014–2015 (Appendix A Figure A2).

**Figure A3:**
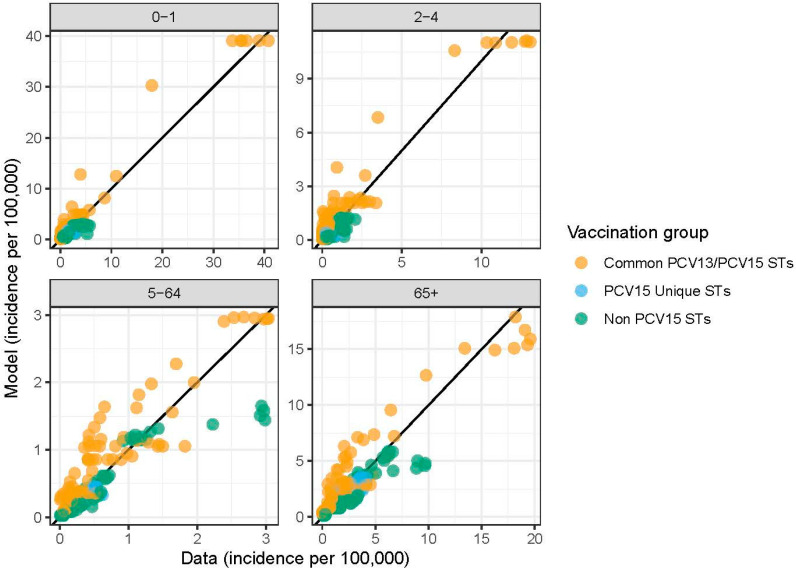
Model validation of the calibrated model comparing the data to the model fit during the calibration period, by age group. Points denote annual and STC-specific incidence and are colored by vaccination grouping. The black line indicates a 1:1 line. The root mean squared error (RMSE) values were 1.47, 0.597, 0.312, and 1.29 for the <2-, 2–4-, 5–64-, and ≥65-year-old age groups. Across all age groups, the RMSE was 1.03.

The root mean squared error (RMSE) was used post hoc to compare model predictions to surveillance data by age, year, and STC, wherein an RMSE closer to zero indicates a better fit while a higher value indicates more error. The RMSE values were 1.47, 0.597, 0.312, and 1.29 for the <2-, 2–4-, 5–64-, and ≥65-year-old age groups (Appendix A Figure A3). Across all age groups, the RMSE was 1.03. A complete description of the model-fitting results and parameter estimates are available in the work by Oidtman et al. 2025 [11].

### 3.2. Projections of IPD in Children Less than Two Years Old

**Figure 1:**
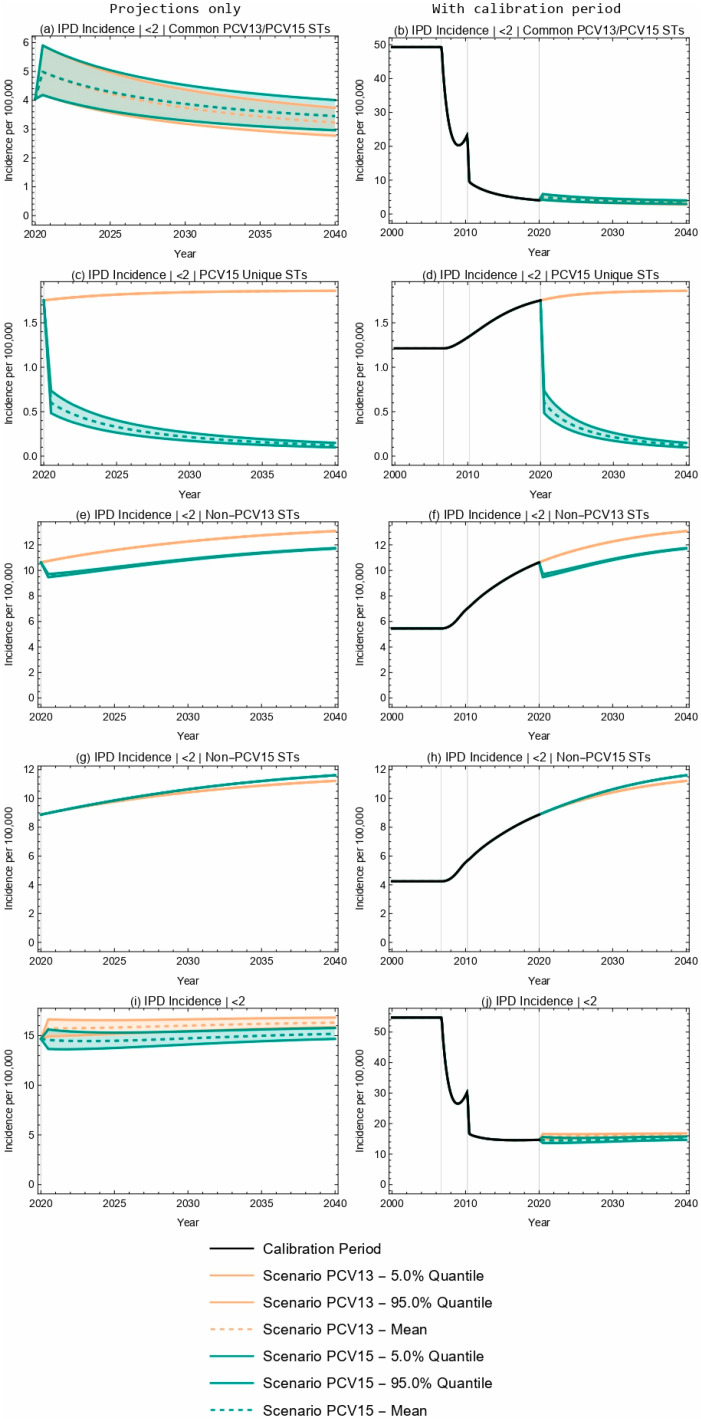
Twenty-year projections of IPD incidence in <2-year-olds by serotype grouping. (**a**,**b**) serotypes common to both PCV13 and PCV15; (**c**,**d**) serotypes unique to PCV15 (i.e., covered by PCV15 but not PCV13); (**e**,**f**) serotypes not covered by PCV13; (**g**,**h**) serotypes not covered by PCV15; (**i**,**j**) all serotypes. The two columns showcase the same results; the left column focuses on the projection period only while the right column includes the calibration period. The first three rows illustrate IPD by ST grouping and the fourth row illustrates overall IPD incidence in <2-year-olds. ST—serotype.

For both PCV13 and PCV15, the model projected an increase in overall IPD incidence in children less than two years old over a 20-year time horizon as a consequence of switching from a 2 + 1 to a 1 + 1 dosing schedule in 2020 (Figure 1). The model outcomes for PCV13 and PCV15 reflect an overlap in projected IPD incidence for shared STCs, with a 14.6–20.0% decline in the incidence of serotypes covered by both PCV13 and PCV15 over the 20-year time horizon following the switch to the reduced dosing schedule (Figure 1a,b, Table 4). IPD incidence due to PCV15-unique STCs declined by 93.1% over the time horizon with the implementation of PCV15, and increased by 6.3% with continued PCV13 vaccination (Figure 1c,d, Table 4). IPD incidence due to serotypes not included in PCV13 increased by 10.3% and 23.0% (Figure 1e,f), and IPD incidence due to serotypes not included in PCV15 increased by 30.7% and 26.4% for PCV15 and PCV13 scenarios, respectively (Figure 1g,h, Table 4). The absolute increase in overall IPD over the time horizon was smaller for PCV15 than for PCV13 (3.5% vs. 11.1%) (Figure 1i,j, Table 4). Sensitivity analyses reflect that results were robust to potential changes in the VE against IPD.

### 3.3. Population-Level Projections of IPD

**Figure 2:**
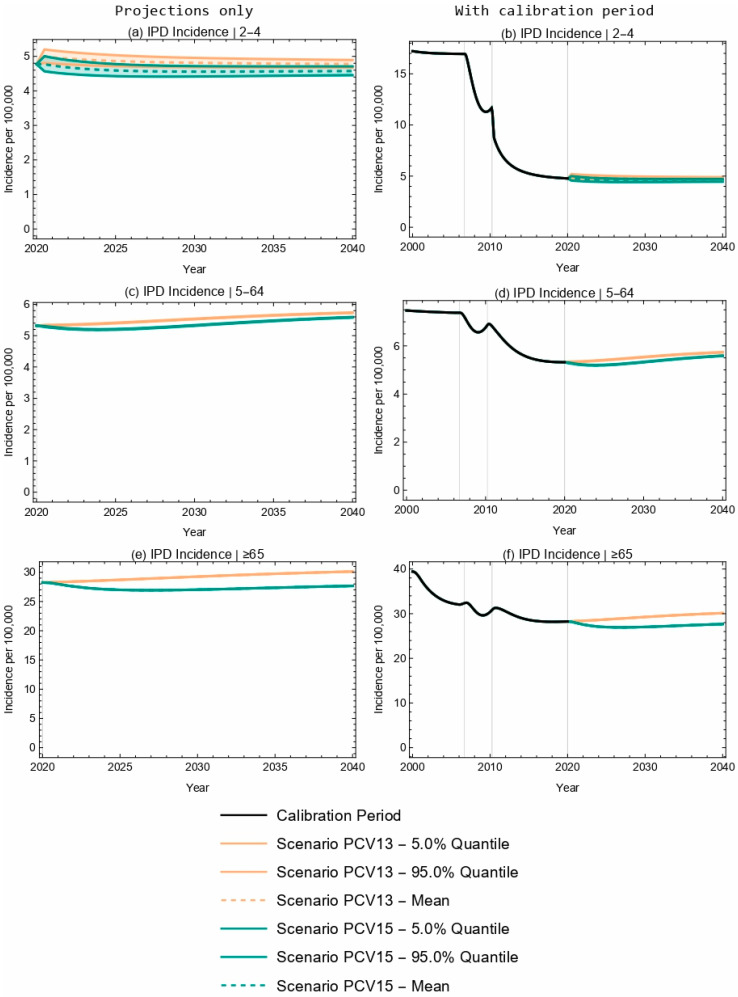
Twenty-year projections of IPD in (**a**,**b**) 2–4-, (**c**,**d**) 5–64-, and (**e**,**f**) ≥65-year-olds. The two columns showcase the same results; the left column focuses on the projection period only, while the right column includes the calibration period. Each row represents a different age group.

**Figure 3:**
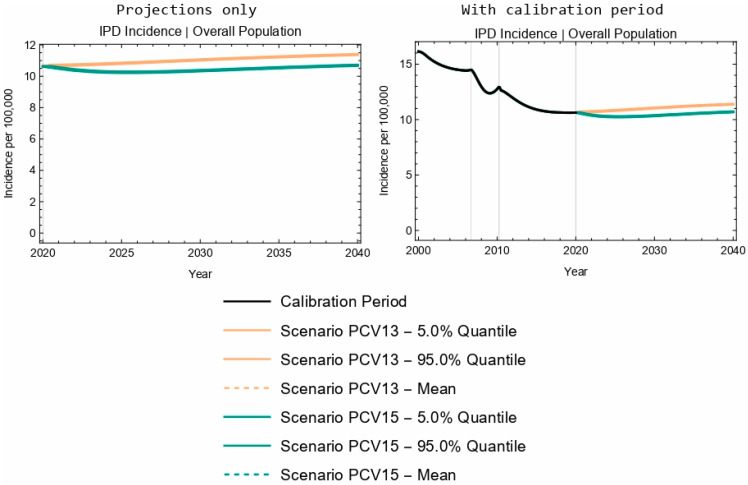
Twenty-year projections of IPD in the entire population. The two columns showcase the same results; the left column focuses on the projection period only, while the right column includes the calibration period.

At the population level, projected overall IPD incidence outcomes for PCV13 and PCV15 overlapped for children aged 2–4 years old over the duration of the 20-year time horizon (Figure 2a,b). Further, the model projected a 4.2% decrease in IPD incidence in the 2–4-year-old age group following the introduction of PCV15, compared with a negligible change in IPD incidence for this same age group with the implementation of PCV13 (Table 5). The model projected a smaller increase in IPD incidence with the introduction of PCV15 when compared with PCV13 for the <2-year-old (3.5% increase vs. 11.2% increase, Table 4) and 5–64-year-old (5.1% increase vs. 7.8% increase) age groups, as well as for the overall population (0.6% increase vs. 7.1% increase, Figure 3 and Table 5). As seen with children <2 years of age, the sensitivity analyses reflect that projected IPD incidence results for all the modeled age groups were robust to potential changes in VE against IPD.

## 4. Discussion

Despite the inclusion of effective PCVs in pediatric vaccination programs, residual pneumococcal disease remains a public health matter in the UK and throughout the world. Here, a previously described and calibrated dynamic transmission model was used to investigate the clinical outcomes resulting from potential reductions in VE with two PCVs (PCV13 and PCV15) when routinely administered in a reduced pediatric dosing schedule (1 + 1) in the UK [10,11]. The model projections predicted that the use of PCV15 would significantly reduce IPD incidence, both in children <2 years of age, due to direct protection, and in older children and adults, due to indirect protection, with respect to the continued use of PCV13. These effects were consistent, regardless of potential changes in VE against disease.

Reductions in IPD in children <2 years old attributed to serotypes unique to PCV15 (22F and 33F) drove PCV15 to avert more disease than PCV13 in model projections. While both PCV13 and PCV15 led to projected decreases in IPD attributed to serotypes covered by both PCV13 and PCV15 in this age group, PCV15 led to a smaller projected decrease when compared with PCV13. Marginal increases in non-PCV15 serotypes were projected. Nevertheless, these differences were insufficient to counteract the overall estimated clinical benefits of pediatric PCV15 vaccination observed in the <2-year-old age group and in the population as a whole. With either vaccine, relative to the current 2019 estimates of IPD incidence, overall IPD incidence was predicted to increase over the 20-year time horizon, though the projected overall increase was predicted to be smaller with the implementation of PCV15 compared to PCV13 vaccination. These results were consistent with the findings of a 2015–2018 global surveillance analysis that found that, across all ages in over 40 countries, the proportion of IPD cases caused by PCV15 serotypes was approximately 4–10% greater than the proportion of IPD cases caused by PCV13 serotypes [39].

In contrast, a recent model by Choi et al. evaluated the implications of introducing higher-valency pediatric vaccines in the UK via the 1 + 1 schedule and concluded that the introduction of PCV15 could result in increased IPD relative to the standard of care of PCV13 [7]. These findings were, in part, a function of this study’s projection assumption on serotype replacement, wherein a decline in the carriage of a VT ST would be met with 100% replacement of carriage with an NVT ST, although this assumption is not substantiated by historical data to date. While it is necessary to make assumptions regarding the level of replacement expected upon the introduction of higher-valency vaccines, assuming 100% replacement may lead to unreasonably high predictions of the disease incidence of NVT STs. In contrast, the current analysis estimated competition between PCV13 and non-PCV13 STs through model calibration. However, there were insufficient data to estimate the competition parameters between PCV15 and non-PCV15 STs; thus, minimal competition between PCV15 and non-PCV15 STs was assumed. In addition, the Choi et al. analysis implemented two separate calibration models for model fitting to historical data, which has the potential to introduce bias and confounding effects due to incompatibilities between the two models [7]. The current analysis employed a single model for calibration and fitting, to ensure compatibility and consistency.

Several simplifying assumptions were made in the current analysis due to model complexity and limited availability of data. A potential reduction in VE against disease resulting from a change to a reduced pediatric vaccination schedule was considered, but there may also be reduced duration of protection and reduced VE against carriage [6]. Reductions in VE against carriage may lead to an increase in VT carriage, which could then increase VT-IPD in pediatric populations due to direct effects and in adult populations due to indirect effects. In addition, consistent with previous modeling studies [7], we did not consider the possibility of immunogenicity creep with the introduction of PCV15. This will be an important area of future research as real-world data become available for expanded-valency PCVs.

Limitations to this analysis are discussed below. First, the model did not account for pneumococcal dynamics during the COVID-19 pandemic. Post-pandemic surveillance calibration data have recently become available, and a recalibration of the model accounting for pneumococcal dynamics during the COVID-19 pandemic is planned as future research. Second, the model is very sensitive to estimates of the calibrated parameters, including VE against carriage. Although this model considered a range of potential reductions in VE against disease that could occur resulting from a change to a reduced dosing schedule, it could not consider potential changes in VE against carriage without conducting a more in-depth parameter identifiability analysis. Investigating the model sensitivity to calibrated parameters, including VE against carriage, is an ongoing area of research. Third, the PSA only considered the VE against disease after the toddler dose (i.e., at the completion of the 1 + 1 dosing schedule). VE in the first year of life (i.e., the VE after receiving the single infant dose in the 1 + 1 schedule) may be more critical to evaluate, given the vulnerability of infants to IPD [5]. However, since the model is not dose-dependent, it could not explicitly predict the effects of two infant doses (in a 2 + 1 schedule) compared to a single infant dose (in a 1 + 1 schedule). Modeling dose-dependent VEs and the potential for breakthrough IPD in infants with a reduced dosing schedule will be an important avenue for future research. Fourth, as there are limited data on real-world estimates of VEs against disease in a 1 + 1 dosing schedule, in part due to the COVID-19 pandemic coinciding with the start of the reduced dosing schedule in the UK, this PSA was instead informed by average changes when moving from a 3 + 1 to 2 + 1 dosing schedule [22] and by assuming parity between PCV13 and PCV15 among common serotypes. Although the assumption of VE parity between PCV13 and PCV15 is critical to this analysis, the assumption is justified by pharmacokinetic studies that predict a comparable efficacy between PCV13 and PCV15 among common serotypes [40,41]. As more estimates of VE against disease and carriage become available, it will be important to incorporate these into model-based predictions. Fifth, to reduce the computational load, this model stratified the population into four age groups, with the 5–64-year-old age group comprising the majority of the population. Since the model did not include restricted carriage transmission from adults to children, this age stratification may have resulted in an under-estimation of disease among all the age groups. While the results of this analysis may be relevant to other countries or regions in a qualitative sense, they are not directly transferable due to geographic variability in serotype distribution. Finally, future work should consider estimating the public health effects associated with other higher-valency pediatric vaccines, including a 20-valent PCV (PCV20).

## 5. Conclusions

Switching from PCV13 to PCV15 for routine pediatric vaccination via the 1 + 1 dosing schedule in the UK would not only further reduce IPD in the pediatric population, but would also lead to population-level reductions in IPD due to indirect protection. Although this model predicted short-term increases in VT-IPD associated with potential reductions in VE against disease in a reduced dosing schedule, the effects were not persistent into the future, and these transient increases were smaller in magnitude with the implementation of PCV15 than the continued use of PCV13. In countries with lower pediatric VCRs, nascent pneumococcal vaccination programs, or uncontrolled VT-IPD, a reduced dosing schedule may lead to more pronounced and persistent increases in VT-IPD.

## Figures and Tables

**Table 1:** Serotype classes (STCs) with respective serotypes (STs) included in each class. Shading indicates inclusion in different vaccines. The vaccination group indicates how serotype classes were grouped for visualization purposes. Note that serotype 3 was included in a stand-alone STC due to evidence of a limited immunogenicity response from vaccines [21].

Serotype Class (STC)	Serotypes Included in Class	Vaccination Group	PCV7	PCV13	PCV15
1	4, 6B, 9V, 14, 18C, 19F, 23F	Common PCV13/PCV15 STs	**-**	**-**	**-**
2	1, 5	Common PCV13/PCV15 STs		**-**	**-**
3	3	Common PCV13/PCV15 STs		**-**	**-**
4	7F, 19A	Common PCV13/PCV15 STs		**-**	**-**
5	6A, 6C ^A^	Common PCV13/PCV15 STs		**-**	**-**
6	22F, 33F	PCV15 Unique STs			**-**
7	9N, 17F, 20	NVTs			
8	8, 10A, 11A, 12F	NVTs			
9	15B	NVTs			
10	15A, 15C, 16F, 23A, 23B, 24F, 31, 35B	NVTs			
11	NVTs ^B^	NVTs			

NVT = non-vaccine type; ST = serotype. ^A^ ST 6C is included in STC 6 due to assumed cross-protection with serotype 6A. ^B^ To simplify the model, the number of STCs was reduced by classifying ST 2 as an NVT. This assumption was anticipated to have minimal impact on the model predictions due to the low prevalence of ST 2.

**Table 2:** Vaccine efficacy values used for the probabilistic sensitivity analysis. A 12.8% average reduction corresponds to the reduction from 0.897 (3 + 1) to 0.782 (2 + 1), as described by Savulescu et al. [22].

STC (STs)	Serotype Grouping	Maximum VE ^A^	Base-Case VE ^B^	Minimum VE ^C^
1 (4, 6B, 9V, 14, 18C, 19F, 23F)	Common PCV13/PCV15 STs	0.96	0.84	0.71
2 (1, 5)	Common PCV13/PCV15 STs	0.84	0.74	0.63
3 (3)	Common PCV13/PCV15 STs	0.41	0.36	0.31
4 (7F, 19A	Common PCV13/PCV15 STs	0.93	0.82	0.69
5 (6A, 6C)	Common PCV13/PCV15 STs	0.96	0.84	0.71
6 (22F, 33F)	PCV15 Unique STs	0.94	0.83	0.70

STC—serotype class; ST—serotype; VE—vaccine effectiveness. ^A^ The maximum VE was assumed to be equal to the VE for the 2 + 1 vaccine schedule. ^B^ The base-case VE was assumed to be 12.8% lower than the VE for the 2 + 1 vaccine schedule. ^C^ The minimum VE was assumed to be equal to 2 × 12.8% = 25.6% lower than the VE for the 2 + 1 vaccine schedule. NOTE: VE for STC 7-11 was assumed to be 0 for PCV7, PCV13, and PCV15 vaccines.

**Table 3:** Projected vaccination scenarios for PCV13 and PCV15 in a 1 + 1 dosing schedule.

Population	Vaccination Age	Vaccine	VCR
Scenario 1			
<2-year-olds	12 weeks, 1 year	PCV13	93.2%
2–64-year-olds	2–64 years at-risk	PPSV23	4.78%
≥65-year-olds	≥65-year-olds	PPSV23	69.2%
Scenario 2			
<2-year-olds	12 weeks, 1 year	PCV15	93.2%
2–64-year-olds	2–64 years at-risk	PPSV23	4.78%
≥65-year-olds	≥65-year-olds	PPSV23	69.2%

**Table 4:** Projected IPD incidence (per 100,000) by serotype grouping in <2-year-olds. IPD incidence at the start of the projection (0 years) and at the end of the 20-year time horizon, with the administration of either PCV13 or PCV15 in the <2-year-old population. The mean projections of IPD (over 100 Latin hypercube samples), the percent change from the start of projections, and the 90% intervals (5% and 95% quantiles) are included. NA values indicate the same value across all PSA realizations, and therefore, there is no informative interval. NA—not applicable.

Serotype Grouping	Initial IPD Incidence at 0 Years	Average IPD Incidence at 20 Years (Change versus Initial Incidence)	90% Interval for IPD Incidence at 20 Years
Pediatric vaccination with PCV13			
Common PCV13/PCV15 serotypes	4.04	3.23 (−20.0%)	(2.78, 3.74)
Unique PCV15 serotypes	1.75	1.86 (+6.3%)	NA
Serotypes not included in PCV13	10.63	13.08 (+23.0%)	NA
Serotypes not included in PCV15	8.88	11.22 (+26.4%)	NA
Overall	14.67	16.30 (+11.1%)	(15.90, 16.80)
Pediatric vaccination with PCV15			
Common PCV13/PCV15 serotypes	4.04	3.45 (−14.6%)	(2.96, 4.01)
Unique PCV15 serotypes	1.75	0.12 (−93.1%)	(0.10, 0.15)
Serotypes not included in PCV13	10.63	11.73 (+10.3%)	(11.71, 11.76)
Serotypes not included in PCV15	8.88	11.61 (+30.7%)	NA
Overall	14.67	15.18 (+3.5%)	(14.70, 15.80)

**Table 5:** Projected IPD incidence (per 100,000) by age. IPD incidence at the start of the projection (0 years) and at the end of the 20-year time horizon, with the administration of either PCV13 or PCV15 in the <2-year-old population. The mean projections of IPD, the percent change from the start of projections, and the 90% confidence intervals (5% and 95% quantiles) are included.

Age Group	IPD Incidence at 0 Years	Average IPD Incidence at 20 Years (Change versus Initial Incidence)	90% Interval for IPD Incidence at 20 Years
Pediatric vaccination with PCV13			
<2	14.67	16.30 (+11.2%)	(15.85, 16.82)
2–4	4.77	4.78 (+0.1%)	(4.67, 4.89)
5–64	5.32	5.73 (+7.8%)	(5.73, 5.74)
≥65	28.25	30.12 (+6.6%)	(30.12, 30.12)
Overall	10.63	11.38 (+7.1%)	(11.36, 11.41)
Pediatric vaccination with PCV15			
<2	14.67	15.18 (+3.5%)	(14.66, 15.76)
2–4	4.77	4.57 (−4.2%)	(4.45, 4.71)
5–64	5.32	5.59 (+5.1%)	(5.58, 5.60)
≥65	28.25	27.67 (−2.1%)	(27.67, 27.67)
Overall	10.63	10.70 (+0.6%)	(10.67, 10.72)

## Data Availability

All the relevant data are within the manuscript and Appendix A. This is a modeling study and, therefore, no primary data were collected in this study. All the inputs were from published literature and included only anonymized data.

## Abbreviations

The following abbreviations are used in this manuscript:

UK	United Kingdom
PCV	Pneumococcal conjugate vaccine
VE	Vaccine effectiveness
IPD	Invasive pneumococcal disease
NIP	National Immunization Programme
PD	Pneumococcal disease
NVT	Non-vaccine type
VT	Vaccine type
NBPP	Non-bacteremic pneumococcal pneumonia
ST	Serotype
STC	Serotype class
HSA	Health Security Agency
VCR	Vaccine coverage rate
PSA	Probabilistic sensitivity analysis
RMSE	Root mean squared error

## Appendix A

**Table A1:** Vaccine efficacy against disease for the <2-year-old age group [22] for a 2 + 1 schedule.

Serotype Class (ST)	VE Against Disease (PCV7)	VE Against Disease (PCV13)	VE Against Disease (PCV15)
1 (4, 6B, 9V, 14, 18C, 19F, 23F)	0.96	0.96	0.96
2 (1, 5)	0	0.84	0.84
3 (3)	0	0.41	0.41
4 (7F, 19A)	0	0.93	0.93
5 (6A, 6C)	0	0.96	0.96
6 (22F, 33F)	0	0	0.94
7 (9N, 17F, 20)	0	0	0
8 (8, 10A, 11A, 12F)	0	0	0
9 (15B)	0	0	0
10 (15A, 15C, 16F, 23A, 23B, 24F, 31, 35B)	0	0	0
11 (NVTs)	0	0	0

VE = vaccine efficacy; NVT = non-vaccine type; ST = serotype.

**Table A2:** Vaccine efficacy against disease for the 2–4-year-old and 5–64-year-old risk groups and the ≥65-year-old population for PPSV23 [23]. Note that, given the lack of real-world evidence for vaccine effectiveness against disease for novel vaccine serotypes (STCs 6–10), we assumed equivalency with previous VTs in adult PCVs.

Serotype Class (ST)	VE Against Disease (PPSV23)
1 (4, 6B, 9V, 14, 18C, 19F, 23F)	0.45
2 (1, 5)	0.23
3 (3)	0.22
4 (7F, 19A)	0.59
5 (6A, 6C)	0
6 (22F, 33F)	0.54
7 (9N, 17F, 20)	0.65
8 (8, 10A, 11A, 12F)	0.57
9 (15B)	0.53
10 (15A, 15C, 16F, 23A, 23B, 24F, 31, 35B)	0
11 (NVTs)	0

VE = vaccine efficacy; NVT = non-vaccine type.

**Table A3:** PCV coverage for the <2-year-old age group [42]. NA—not applicable.

Year	VCR
2005	NA
2006	NA
2007	89.1
2008	90.1
2009	92.9
2010	93.6
2011	94.2
2012	94.4
2013	94.1
2014	93.9
2015	93.5
2016	93.5
2017	93.3
2018	92.8
2019	93.2

VCR = vaccination coverage rate.

**Table A4:** PPSV23 coverage for the ≥65-year-old age group. Data were from 2005–2018. For 2019, we assumed the same coverage as 2018 [43].

Year	VCR
2005	64.4
2006	66.6
2007	69.0
2008	68.2
2009	69.4
2010	70.5
2011	68.3
2012	69.1
2013	68.9
2014	69.8
2015	70.1
2016	69.8
2017	69.5
2018	69.2
2019	69.2

VCR = vaccination coverage rate.

**Table A5:** PPSV23 coverage for the at-risk 2–4- and 5–64-year-old age groups. These calculations assumed that the VCR of clinical risk groups was 49.0% and that the proportion of the 2–64-year-old population that fell within a clinical risk group was 9.7% [43].

Year	VCR
2005–2019	4.78

VCR = vaccination coverage rate.

**Table A6:** Estimated duration of carriage (days) by age group and serotype class [11].

Serotype Class (ST)	0–1	2–4	5–64	65+
1 (4, 6B, 9V, 14, 18C, 19F, 23F)	70.24	30.60	24.94	26.83
2 (1, 5)	13.38	9.58	7.04	6.56
3 (3)	24.60	21.50	39.72	45.50
4 (7F, 19A)	57.98	24.51	21.14	21.45
5 (6A, 6C)	53.60	36.20	24.58	25.84
6 (22F, 33F)	87.56	61.33	46.26	43.10
7 (9N, 17F, 20)	34.73	25.07	18.03	16.58
8 (8, 10A, 11A, 12F)	39.43	26.59	20.86	21.47
9 (15B)	42.24	31.88	21.32	21.75
10 (15A, 15C, 16F, 23A, 23B, 24F, 31, 35B)	36.62	26.04	17.22	17.48
11 (NVTs)	35.26	30.70	29.32	31.21
